# Primary Chest Wall Abscess Mimicking a Breast Tumor That Occurred after Blunt Chest Trauma: A Case Report

**DOI:** 10.1155/2014/620876

**Published:** 2014-02-09

**Authors:** Yusuke Yamaoka, Jun Yamamura, Norikazu Masuda, Hiroyuki Yasojima, Makiko Mizutani, Shoji Nakamori, Toru Kanazawa, Keiko Kuriyama, Masayuki Mano, Mitsugu Sekimoto

**Affiliations:** ^1^Department of Surgery, National Hospital Organization Osaka National Hospital, 2-1-14 Hoenzaka, Chuo-ku, Osaka 540-0006, Japan; ^2^Department of Surgery, Breast and Endocrine Surgery, Sakai City Hospital, 1-1-1 Minamiyasuicho, Sakai-ku, Sakai-shi, Osaka 590-0064, Japan; ^3^Departments of Surgery and Breast Oncology, National Hospital Organization Osaka National Hospital, 2-15, Yamadaoka, Suita-shi, Osaka 565-0871, Japan; ^4^Department of Diagnostic and Interventional Radiology, Osaka University Graduate School of Medicine, 2-1-14 Hoenzaka, Chuo-ku, Osaka 540-0006, Japan; ^5^Department of Radiology, National Hospital Organization Osaka National Hospital, 2-1-14 Hoenzaka, Chuo-ku, Osaka 540-0006, Japan; ^6^Department of Central Laboratory and Surgical Pathology, National Hospital Organization Osaka National Hospital, 2-1-14 Hoenzaka, Chuo-ku, Osaka 540-0006, Japan

## Abstract

Primary chest wall abscess occurring after blunt chest trauma is rare. We present the case of a 50-year-old woman who presented with a swelling in her left breast. The patient had experienced blunt chest trauma 2 months back. Needle aspiration revealed pus formation in the patient's chest. Computed tomography revealed a mass in the lower region of the left mammary gland, with thickening of the parietal pleura and skin and fracture of the fifth rib under the abscess. Following antibiotic administration and irrigation of the affected region, surgical debridement was performed. During surgery, we found that the pectoralis major muscle at the level of the fifth rib was markedly damaged, although the necrotic tissue did not contact the mammary gland. We diagnosed the lesion as a chest wall abscess that occurred in response to blunt chest trauma. Her postoperative course was uneventful. There has been no recurrence for six months after surgery.

## 1. Introduction

Chest wall infections can be categorized as primary and secondary infections, with the former arising spontaneously (primary chest wall abscess) and the latter occurring in response to preexisting disease states or irritation caused by other procedures (secondary chest wall abscess) [1]. We report the case of a 50-year-old woman who developed a primary chest wall abscess with breast tumor—like features that developed after rib fracture caused by blunt chest trauma.

## 2. Case Report

**Figure 1:**
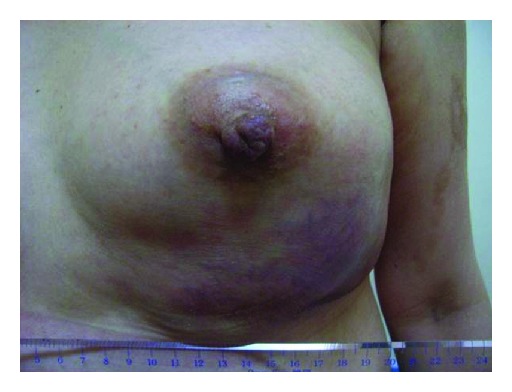
The patient presents with left breast swelling.

**Figure 2:**
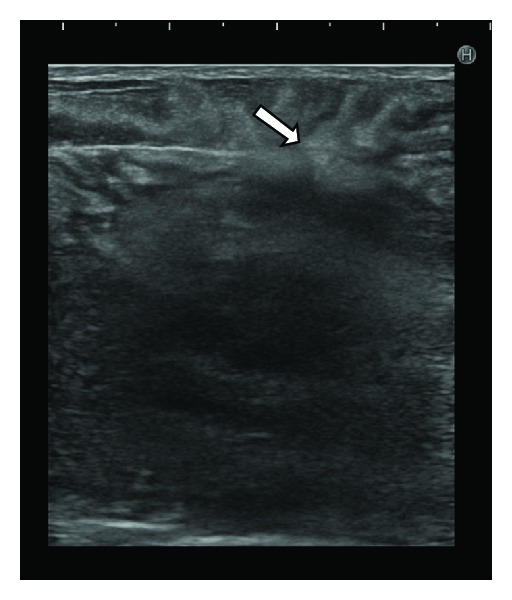
Ultrasound showing a hypoechoic mass with a hyperechoic boundary toward the subcutaneous tissue (arrow).

**Figure 3:**
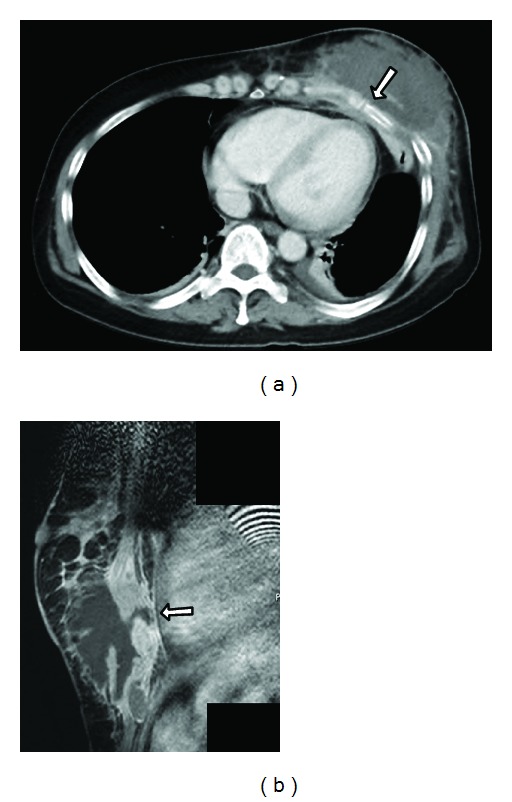
(a) Computed tomography (CT) showing a large mass in the lower region of the left breast. The fifth rib under the mass is fractured (arrow). (b) T1-weighted magnetic resonance imaging reveals contrast enhancement from the border of the mass to the pleura (arrow).

A 50-year-old Japanese female presented at our institute with a swelling in her left breast (Figure 1). She had received a blow to her anterior chest 2 months back, but she did not seek immediate medical consultation at that time. The patient reported no history of major medical diseases such as diabetes mellitus, cardiovascular disease, or renal disease, and she did not smoke, consume alcohol, or take any medications. Physical examination revealed a firm and tender mass in the lower region of the left breast. No lymph nodes were palpable in the bilateral axillary fossa and cervix. Ultrasound revealed a hypoechoic mass with a hyperechoic boundary toward the subcutaneous tissue (Figure 2). Laboratory evaluation revealed a white blood cell (WBC) count of 20,000/*μ*L and a C-reactive protein (CRP) level of 12.3 mg/dL. Serum levels of carcinoembryonic antigen, CA 15-3, and NCC-ST-439 were all within normal limits. Computed tomography (CT) revealed a large mass measuring 10 cm × 10 cm in the lower region of the left breast, with pleural and skin thickening and fracture of the fifth rib under the mass (Figure 3(a)). Contrast-enhanced T1-weighted magnetic resonance imaging with fat suppression revealed a low-signal intensity mass in the lower region of the left mammary gland, and contrast enhancement revealed its border and the adjacent pleura (Figure 3(b)).

Needle aspiration from the mass using an ultrasonic guide revealed pus formation. We suspected a breast abscess or chest wall abscess and instructed that the patient be admitted for treatment. Pus culture revealed the growth of group A *Streptococcus* (GAS; *Streptococcus pyogenes*). Cytology revealed no malignancy.

The patient underwent drainage under local anesthesia on the day of admission, and a cloudy, yellowish-white pus was obtained. An irrigation drain was inserted into the abscess cavity. Antibiotic therapy with an ampicillin-sulbactam combination was administered and saline irrigation (1000 mL/day) via the drain was performed. GAS was susceptible to the ampicillin-sulbactam combination.

**Figure 4:**
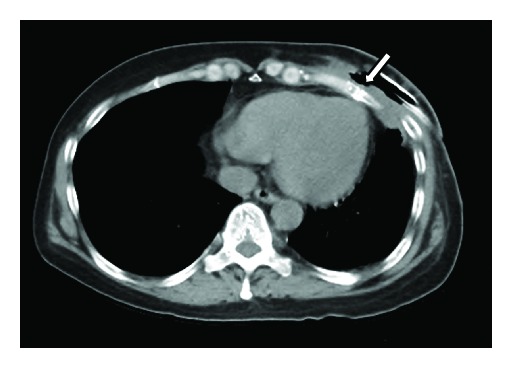
Computed tomography showing that the abscess cavity has shrunk and that the fracture in the fifth rib has healed (arrow).

WBC and CRP levels declined to their normal limits on the 5th day of admission; therefore, antibiotic administration was discontinued. On the 24th day of admission, CT showed that the abscess cavity had shrunk and that the fracture of the fifth rib had healed (Figure 4).

The patient underwent surgical debridement under general anesthesia on the 27th day of admission to reveal the focus of the infection and for removal of the necrotic tissue. During surgery, we found that the pectoralis major muscle at the level of the fifth rib had markedly thinned; this necrotic tissue made no contact with the mammary gland. On the basis of these findings, a final diagnosis of chest wall abscess was made. The patient's postoperative course was uneventful and she was discharged 12 days after surgery. There has been no evidence of recurrence for six months after surgery.

## 3. Discussion

Primary chest wall abscess is a rare clinical entity, occurring as a result of the hematogenous spread of bacterial, fungal, or mycobacterial pathogens. Chest wall abscess occurring because of the spread of infection from the lungs or pleura or that occurring after open trauma or thoracic wall surgery is classified as secondary chest wall abscess. Chest wall abscess can evolve from a simple soft-tissue infection, osteomyelitis of the rib(s), infection of the costochondral junction with destruction of the surrounding ribs, or infection of the sternoclavicular joint [2]. Treatment differs with the extent of infection and includes simple administration of routine antibiotic therapy or multiple and prolonged drainage and complex reconstructive surgeries [1]. The necessity for correct diagnosis cannot be underestimated because fulminant infections can occur in aged or immunocompromised patients if treated incorrectly. Prompt and appropriate surgical management and antibiotic therapy affect the treatment outcome [3].

Our patient developed a chest wall abscess 2 months after receiving a blunt chest trauma, and CT revealed a fracture in her left fifth rib under the abscess. We could not determine the route of entry of GAS into the bloodstream because the patient had been asymptomatic, had received no dental treatment or cut wounds for several months prior to the chest wall abscess, and had no preexisting disease. GAS bacteremia probably resulted from penetration of the skin following minor skin abrasion or trauma [4]. GAS is one of the most common human pathogens, and it can cause a variety of skin and soft-tissue infections, some of which are severe and even life-threatening [5]. We assumed that the patient developed GAS bacteremia in some manner, because of which the soft tissue around the fractured left fifth rib became infected. The fifth rib was located under the breast, so her initial presentation was breast swelling. Inflammatory breast cancer should be considered as a differential diagnosis of such swellings. However, breast cancer in our patient was excluded after the detection of pus and the absence of malignant cells on cytological examination. We also excluded breast abscess because the patient was not lactating, and the absence of inflammation in the left mammary gland was confirmed during surgery.

We performed a PubMed search of the English literature from 1966 to the present using the following keywords: chest, abscess, and blunt trauma. We also retrieved cases from the relevant reference lists. There were seven previous reports of primary chest wall abscess occurring after blunt chest trauma (Table 1) [2, 6–11]. Fractures were found in 4 of 8 cases. In five cases, the abscess appeared more than 2 months after the blunt trauma for reasons that remained unclear. The primary focus of infection remained unclear in six cases, and in five of these six cases, the pathogenic bacteria were indigenous skin bacteria. The treatment differed depending on the extent of infection.

In summary, we report a case of primary chest wall abscess that developed after blunt chest trauma and initially mimicked a breast tumor. Because abscess formation was behind the mammary gland, breast tumor should be considered a differential diagnosis. Although the precise mechanism of this infection remained unclear, the patient's condition improved without extensive debridement. If we had performed surgical debridement first, the patient would have been discharged earlier. In conclusion, this report suggests that chest wall abscess should be considered as a differential diagnosis when a patient presents with breast swelling and reports a history of blunt chest trauma.

## Figures and Tables

**Table 1:** Reported cases of primary chest wall abscess occurring after blunt chest trauma.

Reference	Age	Sex	Injured location	Fracture	Time from trauma to onset	Causative pathogen	Primary focus of infection	Surgical intervention	Outcome
Caruana and Swayne [6]	37	M	7th rib	−	2 months	*Salmonella typhi *	Enteritis	Surgical debridement with resection of the ribs	Recovered
Hananel et al. [7]	37	M	Sternum	−	2 months	*Salmonella typhi *	Unknown	Only pus drainage	Recovered
Gregory [8]	37	M	Manubrium, sternum, and two ribs	+	14 days	*Staphylococcus aureus *	Unknown	Surgical debridement with resection of the manubrium and the sternum	Recovered
Jayle et al. [9]	14	M	Sternum	−	3 years	*Staphylococcus aureus *	Unknown	Surgical debridement with resection of the sternum	Recovered
Gilart et al. [10]	62	n/m	5–9th ribs	+	5 days	*Staphylococcus aureus *	Unknown	Surgical debridement	Recovered
Sakran and Bisharat [2]	65	F	4th rib	−	2 months	*Escherichia coli *	Urinary tract infection	Only pus drainage	Recovered
Ichimura et al. [11]	15	M	Sternum	+	19 days	*Staphylococcus aureus *	Unknown	Only pus drainage	Recovered
Present case	50	F	5th rib	+	2 months	*Streptococcus pyogenes *	Unknown	Surgical debridement	Recovered

M: male; F: female; n/m: not mentioned.
